# USP19 deubiquitinates HDAC1/2 to regulate DNA damage repair and control chromosomal stability

**DOI:** 10.18632/oncotarget.11116

**Published:** 2016-08-08

**Authors:** Min Wu, Hai-qing Tu, Yan Chang, Bo Tan, Guang Wang, Jie Zhou, Li Wang, Rui Mu, Wei-na Zhang

**Affiliations:** ^1^ State Key Laboratory of Proteomics, National Center of Biomedical Analysis, Institute of BasicMedical Sciences, Beijing 100850, China; ^2^ Beijing Institute of Biotechnology, Beijing 100071, China; ^3^ Department of Radiation Oncology, National Cancer Center/Cancer Hospital, Chinese Academy of Medical Sciences and Peking Union Medical College, Beijing 100021, China

**Keywords:** USP19, HDAC1/2, DNA repair, genome stability

## Abstract

Excessive accumulation of DNA damage will generate chromosome stress, leading to various chromosome abnormalities such as chromatin bridge and result in genomic instability. Orchestra procession and regulation of DNA damage repair are vital for keeping genome stability. Despite of the key role of HDAC1/2 in double strand break (DSB) repair, the regulation for their mode of action is less well understood. In this study, we found that deubiquitination enzymes USP19 physically interacts with HDAC1/2 and specifically regulate their K63-linked ubiquitination, which might be crucial for regulation of HDAC1/2 activity in DNA damage repair. Notably, we found that USP19 trans-locate into nucleus upon IR irradiation and is indispensable for normally DNA damage response. In addition, we showed that USP19 play critical role in preventing anaphase bridge formation through regulating DNA damage repair process. Furthermore, the expression level of USP19 is commonly lower or deleted in several types of tumor. These results indicated that USP19 is a key factor in modulating DNA damage repair by targeting HDAC1/2 K63-linked ubiquitination, cells with deletion or decreased expression of USP19 might cause genome instability and even contribute to tumorigenesis.

## INTRODUCTION

The exposure of cells to various genotoxic stresses will lead to DNA damage which would jeopardize the genome integrity. DNA damage response (DDR) triggers DNA repair to prevent genome instability [1]. Generally, double-strand breaks (DSBs) is the most consequential types of DNA damage and are mainly repaired by either homologous recombination (HR), which is limited to the S and G2 phase of cell cycle, or non-homologous end-joining (NHEJ), which operates throughout the cell cycle [2]. Anaphase bridge, which is usually happened in the case of genomic instability will be induced if double-strand breaks (DSB) could not be normally repaired due to some kind of defect in DNA repair [3, 4]. Accumulation of DNA damage in cells would lead to chromosome mis-segregation, which may entail chromatin/anaphase bridges, prevent normal cytokinesis and finally high rates of chromosomal mis-segregation would cause chromosome instability(CIN), which is a common characterize for majority of human cancer [5, 6]. Thus, DNA repair pathway which is tightly controlled by several important factors is vital to maintain genome stability.

Histone deacetylases (HDAC) are a class of enzymes that remove acetyl groups from an N-acetyl lysine amino acid on a histone, allowing the histones to wrap DNA more tightly. This epigenetic modulation has been shown to resulting in the formation of an inactive chromatin structure that represses DNA transcription. There are four classes of HDACs in human cells including 18 known HDACs. HDAC1-3 and HDAC8 belongs to Class 1 HDACs, which are ubiquitously expressed and show the strongest enzyme activity [7]. By targeting histone or other non-histone proteins, HDACs play critical roles in cellular growth, differentiation, apoptosis, and tumorigenesis [8]. Recent study have demonstrated that human deacetylases HDAC1 and HADC2 play critical role in DNA-damage response by promoting DSB repair, especially for NHEJ repair, through regulating histone H3K56 acetylation. Depletion of HDAC1 and HDAC2 in cells impairs DNA repair and then leads to sustained DNA-damage signaling. Consistently, these cells are hypersensitive to DNA-damaging agents [9]. Except for HDAC1/2, another HDAC deacetylases family member, SIRT1 is also reported to be recruited to DSB and primes the cellular response to DNA damage by stimulating the activity of ATM and HDAC1 [10]. Although HDAC is crucial for efficient DNA damage repair, the precise mechanism for HDAC regulation upon DNA damage remains poorly understood.

Protein modification by ubiquitin controls numerous important cellular processes such as transcription, DNA repair and cell cycle progression [11]. Like most posttranslational modification, ubiquitination is also a reversible process performed by deubiquitination enzymes (DUB), of which only a few has been functionally characterized. Despite the vast understanding of the key role for protein ubiquitination in DNA damage response [12–14], whether deubiquitination participates in this procedure is largely unknown. Ubiquitin-specific processing proteases (USPs) are recognized as the largest class of DUB by the presence of a core catalytic domain of ~450 amino acids separated by cysteine and histidine box [15]. USP19 belongs to USPs family and is shown to regulate cell cycle progression, cell differentiation, hypoxia response, apoptosis and endoplasmic-reticulum-associated degradation (ERAD) by targeting different substrate such as p27, HIF1α and cIAP for deubiquitination [16–18]. Until now, little is known about the regulation of USP19 in DNA damage response and its role in DSBs repair.

In this study, by using siRNA library screening for genes that may affect mitosis progression through time-lapes, we found USP19 knock down leads to obvious chromosome mis-segregation. Further analysis showed that USP19 play critical role in preventing anaphase bridge formation through regulating DNA damage repair process. Importantly, we found that USP19 binds to HDAC1/2 and specifically regulating their K63-linked ubiquitination, which might be crucial for regulation of HDAC1/2 activity in DNA damage repair. Furthermore, the USP19 gene is commonly deep deleted in several types of tumor samples. These results indicated that USP19 is a key factor in modulating DNA damage repair by targeting HDAC1/2 K63-linked ubiquitination and is vital for maintaining genome stability.

## RESULTS

### Knockdown USP19 induces the formation of anaphase bridge

**Figure 1:**
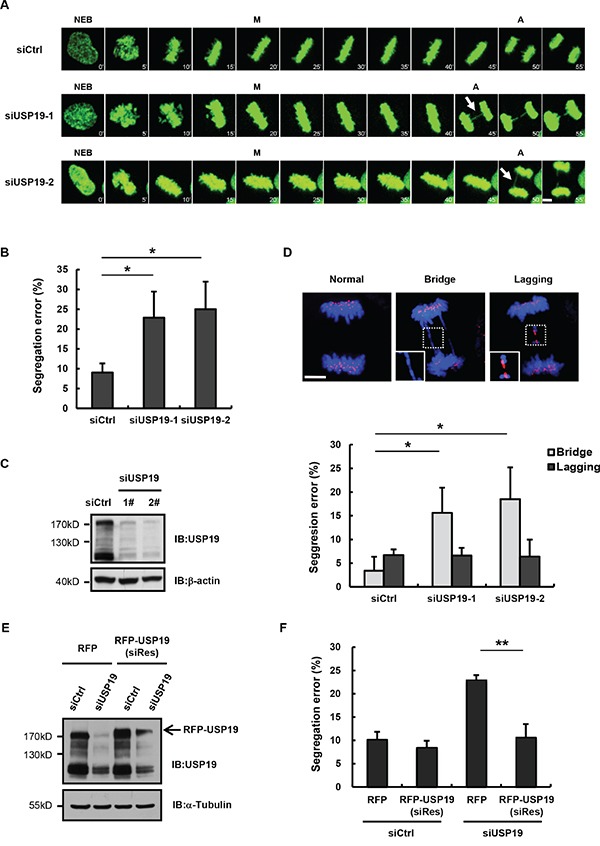
Knockdown USP19 induces the formation of anaphase bridge **A**. Selected frames from time-lapse movies of representative HeLa/GFP-H2B cells transfected with control siRNA (siCtrl) or USP19 siRNA (siUSP19). Arrows denote segregation error. The time on the images is in minutes. NEB, nuclear envelope breakdown; M, metaphase; A, anaphase. Scale bar, 5 μm. **B**. The percentage of segregation errors in control and USP19 knockdown cells during anaphase. Data are representative of three independent experiments, error bars indicate S.D. **C**. Immunoblot analyzes knockdown efficiency of USP19. **D**. HCT116 cells were transfected with control or USP19 siRNA, then synchronized in mitosis by thymidine block and release. The cells through mitosis were immunostained with anti-centromere antibodies (ACA; for kinetochores; red) and DAPI (for chromosomes; blue). Upper panels shows example images of normal segregation, anaphase bridges and lagging chromosomes in anaphase. Scale bar, 5 μm. Lower panels is quantification of mitotic cells with anaphase bridges or lagging chromosomes in control and USP19 knockdown cells. *p<0.05, **p<0.01. **E, F**. Complementation of RFP-USP19 in knockdown HeLa/GFP-H2B cells rescues chromosome segregation errors. The USP19 knockdown cells were transfected with USP19 siRNA-resistant expression construct or RFP vector. (E) The expression of siRNA-resistant RFP-USP19 was detected by Immunoblot. (F) The data show the percentage of mitotic cells with segregation errors during anaphase in RFP-positive cells, data are shown as mean ± S.D. *p<0.05, **p<0.01.

Mammalian cells that experience chromosome mis-segregation, such as formation of anaphase bridges, usually generate daughter cells with aneuploidy, is largely known as a hallmark of cancer. In a separate mitosis screening study, we found that USP19 knockdown in HeLa cells could result in obvious increase of chromosome segregation errors (from 8% to 25%), which could be confirmed by two separate USP19 siRNA (Figures 1A-1C). This segregation error seems like anaphase bridge formation, since we found that a DNA bridge connects homologous chromosomes. To further confirm these results and clarify what types of error was induced by USP19 known down, HCT116 cells were transfected with control or USP19 siRNA and then stained for kinetochore by using anti-centromere antibody (ACA) through immunofluorescence. Aberrant chromosome segregation, such as chromosome bridge and lagging chromosome (Figure 1D), was counted in cells after the anaphase onset. Although similar level of chromosome lagging (about 7%) occurred in control and USP19 knock down cells, an obvious increase of anaphase bridge was detected in cells with USP19 knock-down (Figure 1D). To rule out off-target effects of USP19 siRNA, HeLa/GFP-H2B cells were co-transfected with red fluorescent protein-tagged siRNA-resistant wild-type USP19 or control vector together with USP19 siRNA (Figure 1E). The chromosome segregation errors induced by USP19 knockdown was reversed by the expression of siRNA-resistant wild-type USP19 (Figure 1F). These data indicate that knock down of USP19 could induce chromosome bridge formation, suggesting USP19 might be involved in preventing this type of chromosome segregation error.

### Cells depleted with USP19 show accumulation of DNA-damage

**Figure 2:**
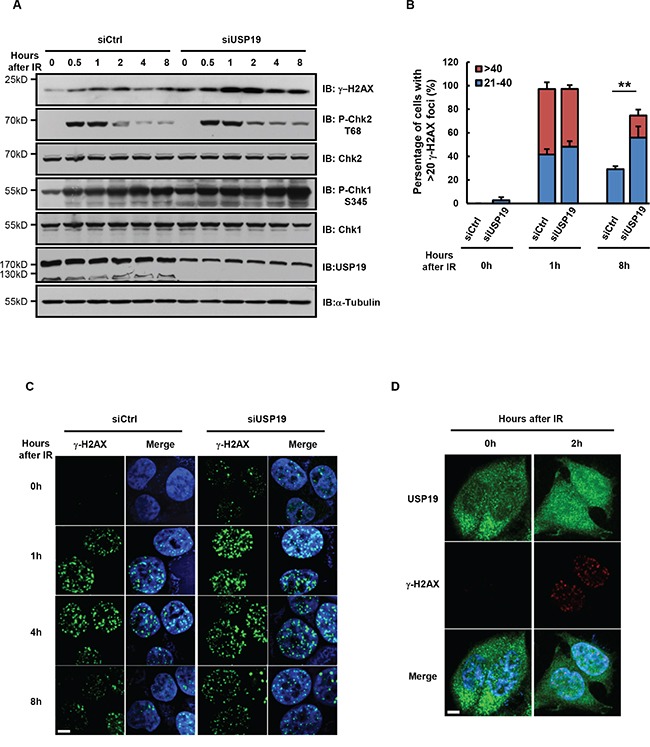
Cells depleted with USP19 show accumulation of DNA-damage **A-C**. HCT116 were transfected with control or USP19 siRNA and irradiated with 5Gy γ-ray. At various time points after irradiation, (A) immunoblot analysis were performed to detect the level of γ-H2AX, phosphorylation of Chk1(P-Chk1 S345), phosphorylation of Chk2(P-Chk2 T68), Chk1, Chk2 and USP19; (B) Irradiated cells were immunostaining with γ-H2AX antibody (green) and DAPI (blue), fraction of cells with the noted numbers of γ-H2AX foci per cell were quantified, data are shown as mean ± S.D; *p<0.05, **p<0.01 (C) representative images of γ-H2AX foci. Scale bar, 5 μm. **D**. USP19 localization after irradiation. Control cells and USP19 knockdown cells were irradiated with 5Gy γ-ray, follow by immunostaining with USP19 antibody (green), γ-H2AX antibody (red) and DAPI (blue). Scale bar, 5 μm.

It has been known that different segregation error usually results from defects in different cell cycle stage. In most cases, chromosome lagging arises from defect in mitosis, while chromosome bridge formation are generally due to defects in pre-mitosis [19, 20]. Forcing cells with damaged DNA into mitosis causes severe chromosome segregation defects, including chromosomal bridges. To further examine the detailed mechanism for USP19 regulation in anaphase bridge formation, we next detected whether USP19 is involved in DNA damage response. As shown in Figure 2A, DNA damage response is triggered in HCT116 cells by IR irradiation, the DNA damage marker γ- H2AX, is rapidly accumulated upon irradiation and then decreased gradually after DNA repairing. Notably, we found the γ- H2AX level was much higher in USP19 knock down cell compared to control cells. Consistently, CHK1/2 activity seems could not be lower down in USP19 knock down cells, indicating that DNA damage constitutively exist in these cells. To further confirm the function of USP19 in DNA damage, immunofluorescence assay was also performed to detect γ- H2AX foci formation in control or USP19 knockdown cells. Similarly, in control cells, γ- H2AX foci was obviously induced at 1 hours after IR irradiation and gradually decreased because of DNA repair. However, when USP19 was knocked down, the accumulated γ- H2AX foci could not disappear efficiently, indicating a defect of DNA repair in USP19 knockdown cells (Figure 2B and 2C). In order to examine what happened to USP19 in response to DNA damage, we detected USP19 localization and found that majority of USP19 trans- located into nucleus upon IR irradiation (Figure 2D), which provide a spatial possibility for USP19 to involve in DNA damage related event. These results suggest that USP19 might plays critical role in DNA damage repair, depletion of USP19 could lead to accumulated DNA damage and then result in chromosome mis-segregation.

### USP19 interacts with HDAC1 and HDAC2

**Figure 3:**
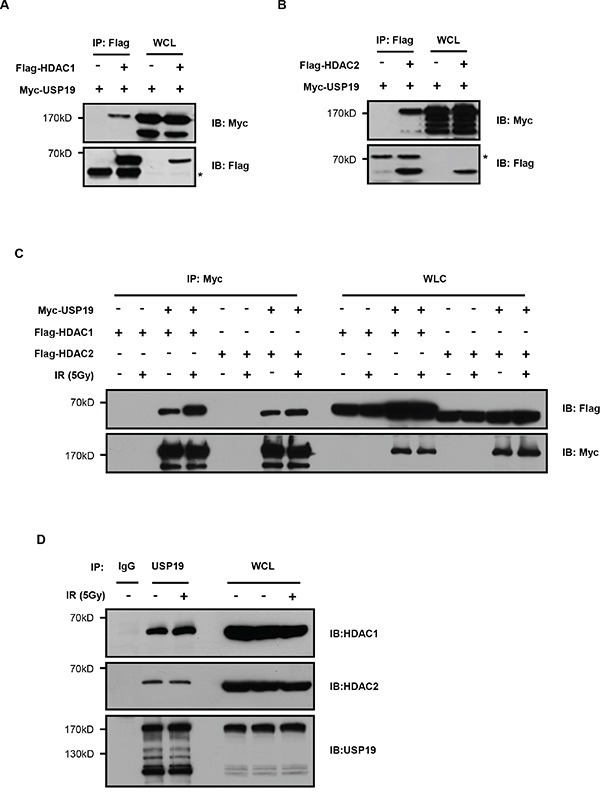
USP19 interacts with HDAC1 and HDAC2 **A**. HEK293T cells were co-transfected with Flag-HDAC1 and Myc-USP19. Cell lysates were subjected to immunoprecipitated (IP) with anti-Flag, and immunoprecipitants or the whole-cell lysates (WCL) were analyzed by immunoblotting (IB) with anti-Myc and anti-Flag. **B**. HEK293T cells were cotransfected with Flag-HDAC2 and Myc-USP19. Cell lysates were subjected to immunoprecipitated with anti-Flag, and immunoprecipitants or the WCL were analyzed by immunoblotting with anti-Myc and anti-Flag. **C**. HCT116 cells were cotransfected as indicated. Two hours after irradiated with 5Gy γ-ray, cell lysates were immunoprecipitated by using Myc antibody, and the bound proteins were analyzed with Flag antibody. **D**. Two hours after irradiated with 5Gy γ-ray, HCT116 cell lysates were immunoprecipitated by using USP19 antibody, and the bound proteins were analyzed with HDAC1/2 antibody.

To further explore the detailed mechanism for USP19 regulation on DNA damage repair, we identified USP19 binding proteins by using an immunoprecipitation assay and data was analyzed by mass spectrometry (unpublished data). Of all the binding proteins, we found USP19 could interact with HDAC1 and HDAC2, which have been reported to play important roles in DNA damage repair. To further confirm the interaction between USP19 and HDAC1/2, we transfected Flag tagged HDAC1 or HDAC2 together with Myc tagged USP19 into HEK293T cells and co-immunoprecipitaion experiment was performed. Immunoblot analysis showed that both HDAC1 and HDAC2 could bind to USP19 (Figure 3A and 3B). In addition, we also detected the association between HDAC1/2 and USP19 upon DNA damage both in exogenous and endogenous station. Interestingly, as shown in Figure 3C and Figure 3D, we found much stronger binding of USP19 to HDAC1 in response to IR irradiation, further suggesting the HDAC-USP19 complex was involved in DNA damage response.

### USP19 specifically deubiquitinates HDAC1/2 for their K63-linked ubiquitination

**Figure 4:**
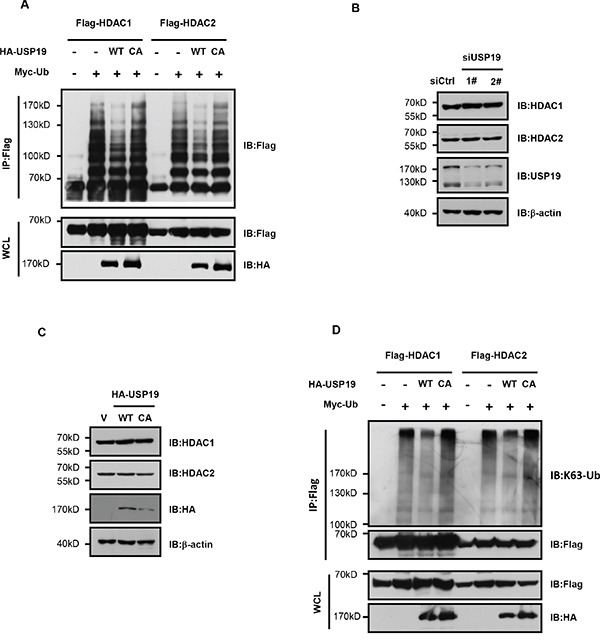
USP19 specifically deubiquitinates HDAC1/2 for their K63-linked ubiquitination **A**. HEK293T cells were transfected with Myc-tagged Ubiquitin (Myc-Ub), Flag-HDAC1, Flag-HDAC2, HA-USP19 WT, HA-USP19 CA as indicated. Cell lysates were extracted under denaturing conditions (95°C, 1% SDS) and immunoprecipitated with anti-Flag, immune complexes were immunoblotted with anti-Flag. **B**. Seventy-two hours after Control or USP19 siRNA transfection, protein levels of HDAC1, HDAC2 and USP19 were assessed by Immunoblot. **C**. HA-USP19 WT, HA-USP19 CA or HA vector was transfected into cells. Seventy-two hours after transfection, protein levels of HDAC1, HDAC2 and HA-USP19 were assessed by Immunoblot. **D**. 293T cells were transfected with Myc-Ub, Flag-HDAC1, Flag-HDAC2, HA-USP19 WT, and HA-USP19 CA as indicated. Cell lysates were extracted under denaturing conditions (95°C, 1% SDS), then immunoprecipitated with anti-Flag and immunoblotted with linkage-specific antibodies recognizing K63-linked polyubiquitin.

To further investigate the potential role of USP19 on HDAC regulation, we next wondered whether it could modulate HDAC ubiquitination since USP19 is largely known as a deubiquitination enzyme. HEK293T cells were transfected with HDAC1 or HDAC2 together with wild type of USP19 or USP19 CA mutant in which the active site Cys-506 was mutated to Ala to block the activity of deubiquitinase USP19. HDAC protein was immunoprecipitated and ubiquitination levels were analysed by immunoblotting. As shown is Figure 4A, the ubiquitination for both HDAC1 and HDAC2 was dramatically dropped down in USP19 transfected cells. However, expression of USP19 CA mutant failed to show any effect on HDAC ubiquitination. As early reports have showed that some HDAC family members could be degraded by K48-linked ubiquitination, we next investigated whether USP19 could regulate HDAC1/2 stability. Surprisingly, we found that neither USP19 knockdown nor its overexpression could infiuence HDAC1/2 stability (Figure 4B and 4C), indicating that USP19-mediated deubiquitination of HDAC1/2 does not affect their degradation. Consistently, little difference for HDAC1/2 ubiquitination between control cells and USP19 overexpression cells was observed if we use K48-linked ubiquitination- specific antibody (Supplemental data). These data indicate that USP19 could not remove the K48-linked ubiquitination in HDAC1/2. Importantly, we found that the K63-linked ubiquitination level for both HDAC1 and HDAC2 could barely be detected in the presence of USP19 but not USP19 CA mutant, suggesting USP19 could specifically deubiquitinate HDAC1/2 for their K63-linked ubiquitination (Figure 4D).

### USP19 is required for NHEJ-mediated DNA repair

**Figure 5:**
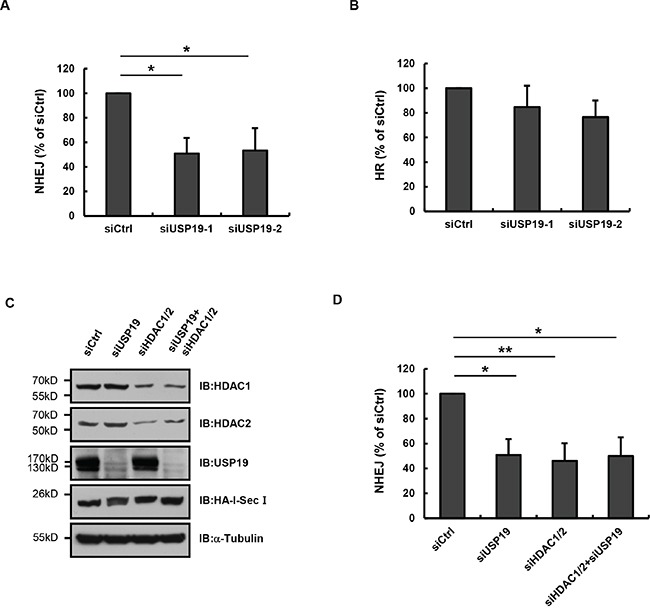
USP19 is required for in NHEJ-mediated DNA repair **A**. EJ5-GFP U2OS cells were cotransfected with control or USP19 siRNA and HA-I-SceI. Cells were collected and analyzed for the proportion of GFP-positive cells by flow cytometric. The NHEJ efficiency presented as relative quantification compared with control siRNA. Data are shown as mean ± S.D. *p<0.05, **p<0.01. **B**. DR-GFP U2OS cells were cotransfected with control or USP19 siRNA and HA-I-SceI. Cells were collected and analyzed for the proportion of GFP-positive cells by flow cytometric. The HR efficiency presented as relative quantification compared with control siRNA. Data are shown as mean ± S.D. **C**. EJ5-GFP U2OS cells were cotransfected with HA-I-SceI and siRNAs as indicated. Protein levels of HDAC1, HDAC2, USP19 and HA-I-SceI were assessed by Immunoblot. **D**. EJ5-GFP U2OS cells were cotransfected with HA-I-SceI and siRNAs as in C. Cells were collected and analyzed for the proportion of GFP-positive cells by flow cytometric. The NHEJ efficiency presented as relative quantification compared with control siRNA. Data are shown as mean ± S.D. *p<0.05, **p<0.01.

Our previous data have demonstrated that USP19 is required for normally DNA damage response, since it binds to HDAC1/2 and regulate their ubiquitination, we next wondered whether USP19 was involved in regulating DNA damage repair. As reported by Stephen P Jackson et.al, HDAC1 and HDAC2 play critical roles in DNA damage response to promote NHEJ repair [9], we next tested whether USP19 also contributes to the process of NHEJ. By using the GFP reporter system, which is revealed by random-plasmid integration as well as I-Sce1-based homologous recombination, we monitored the percentage of cells which can be able to induce NHEJ or HR repair and found that USP19 knock down could definitely lead to a substantial defect in NHEJ but slight reduce in HR (Figure 5A and 5B). These data are consistent with the results that HDAC1 and HDAC2 are required for efficient DNA repair, particularly through NHEJ. Furthermore, we detected NHEJ repair in cells depleted USP19 and HDAC1/2 (knock-down efficiency was shown in Figure 5C) and found that the percentage of cells which could be able to complete NHEJ repair did not drop down much more in USP19 and HDAC1/2 double knock down cells (5D), indicating that USP19 and HDAC1/2 work in the same pathway, which further supporting our hypothesis that USP19 participates in DNA damage repair through regulating HDAC1/2.

### Expression state of USP19 in human tumor samples

**Figure 6:**
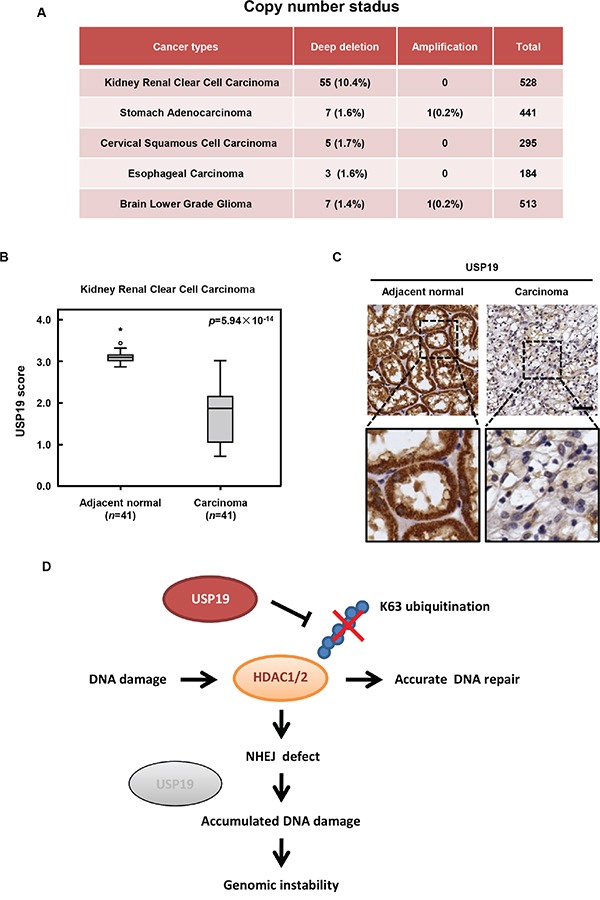
USP19 expression in human cancers and schematic presentation roles of USP19 in DNA damage response **A**. Copy number status of USP19 in various types of human tumor samples from the database of cBioportal for cancer genomics ( http://www.cbioportal.org/index.do. Data source which contains more than 150 clinical samples that are mostly based on TCGA were analyzed, including kidney renal clear cell carcinoma (TCGA provisional), stomach adenocarcinoma (TCGA Nature, 2014), cervical squamous cell carcinoma (TCGA provisional), esophageal carcinoma (TCGA provisional) and brain lower grade glioma (TCGA provisional). **B**. The tissue array of kidney renal clear cell carcinoma was performed by immunohistochemistry with anti-USP19 antibody. USP19 expression was plotted using the score as described in the ‘Materials and Methods’ section. Outliers are indicated by open circles, extremes by asterisks. **C**. Representative images from immunohistochemical staining of USP19 in adjacent normal tissue and kidney renal clear cell carcinoma; scale bar, 50μm. **D**. Schematic presentation roles of USP19 in DNA damage response. In response to IR irradiation, USP19 translocate into nucleus and physically interacts with HDAC1/2 for regulating their K63-linked ubiquitination and involves in NHEJ mediated DNA repair. Due to the defect in DNA repair, cells with USP19 depletion showed accumulated DNA damage which would lead to the formation of chromosome error and finally contribute to genomic instability and even tumorigenesis.

As to its essential role in DNA repair and in maintaining chromosome stability, USP19 might be critical for cells to prevent genome instability and its deletion might be contributed to tumorigenesis. So we next investigated whether deregulation of USP19 expression is associated with human cancers and checked its copy number state in various types of human tumor samples from the database of cBioportal for cancer genomics (http://www.cbioportal.org/index.do). In order to ensure the reliability of data analysis, we only chose the data source which contains more than 150 clinical samples that are mostly based on TCGA. The result showed that 1%-12% deep deletion of USP19 is observed in several different tumors types, including kidney renal clear cell carcinoma (TCGA provisional), stomach adenocarcinoma (TCGA Nature, 2014), cervical squamous cell carcinoma (TCGA provisional), esophageal carcinoma (TCGA provisional) and brain lower grade glioma (TCGA provisional) (Figure 6A). By using tissue array of kidney renal clear cell carcinoma, which rank first in deep deletion rate of USP19 showed in Figure 6A, we further confirmed that USP19 protein level significantly decreased in human kidney renal clear cell carcinoma compared with adjacent normal tissues (Figure 6B and 6C), suggesting that depletion or lower expression of USP19 might be involved in such types of tumorigenesis.

## DISCUSSION

Faithful chromosome segregation is critical for preventing genome integrity. It has been shown that certain errors, such as the mis-regulation of spindle-assembly checkpoint (SAC) activation in mitosis usually cause chromosome mis-segregation, which majorly in the form of chromosome lagging. [21, 22]. However, more and more studies are providing evidences that pre-mitotic replication stress or other double strand DNA damage will generate chromosome stress and finally lead to various chromosome abnormalities, including the formation of chromatin bridge and acentric fragment [19, 20]. Defect in DNA damage repair could result in persisting DSB, the sequelae of the unrepaired DSB could entail chromosome bridges. Thus, identification of novel factors that play critical roles in DNA damage repair might provide important insight for our understanding of chromosome stability regulation and inform new approaches for preventing chromosome mis-segregation and genome instability.

It has been becoming increasingly clear that histone modification such as acetylation, phosphorylation and ubiquitylation is vital for regulating properties of chromatin and thus affect DNA-based processes, such as DNA damage response [23, 24]. Because of the precisely control on histone acetylation, the enzymes histone deacetylases (HDAC) is more and more implicated in regulating DNA damage response. HDAC1 and HDAC2 are shown to be recruited to DNA damage site and is critical for effective DNA repair, especially in NHEJ by modulating H3K56 and H4K16 acetylation. Until now, although the K48-linked ubiquitination of HDAC1/2 had been reported by some groups [25], little is known about the regulation of K63-linked ubiquitination of HDAC1/2. Particularly, the manner of HDAC1/2 regulation in DNA damage is less well understood. In this study, we found that deubiquitination enzymes USP19 translocate into nucleus upon DNA damage treatment and physically interacts with HDAC1/2 for regulating the K63-linked ubiquitination of HDAC1/2. We also showed that USP19 was essential for NHEJ repair. Knockdown of USP19 leads to obvious reduction of NHEJ and cells with USP19 depletion showed much more accumulation for DNA damage foci compared to control cells, indicating USP19 is indispensable for normally DNA damage response. The results obtained in our study are in accordance with the role of HDAC1/2 in DNA damage repair. Overall our data suggest that USP19 primes the cellular repair in response to DNA DSB by regulating HDAC1/2 through controlling its ubiquitination. Due to their defect in DNA repair, cells with USP19 depletion showed accumulated DNA damage and chromosome error, which might be contribute to genomic instability and even tumorigenesis (Figure 6D).

Ubiquitination of many key factors involved in DNA damage response is essential for damage sensing, signal transduction and DNA repair. Until now, most of the known regulators that participate in ubiquitination modulation are E3 ligases, like RNF8 et.al [26], whether any deubiquitination enzymes also involve in DNA damage response remains poorly understood. Here we reported that deubiquitination enzyme USP19 plays an important role in DNA damage repair through deubiquitinating HDAC1/2. The results that USP19 is deep deleted or down-regulated in various human cancers further strengthen the possibility that USP19 might function as a tumor suppresser by facilitating DNA damage repair and protecting chromosome stability. Despite the recent progress of elucidating USP19 function in muscle cell differentiation or development, much more potential roles of USP19 in tumorigenesis are needed to be explored in further study.

## MATERIALS AND METHODS

### Antibodies and reagents

Rabbit anti-USP19 (A301-586A, 1: 1000) was from Bethyl Laboratories Inc. (Montgomery, AL, USA); mouse anti-γH2AX (Ser139) (05-636, 1: 5000 for Immunoblot, 1: 500 for immunostaining) was from Merck-Millipore (Boston, MA, USA); human anti-ACA (# 15-234-0001, 1: 200) was from Antibodies Inc. (Davis, CA, USA); rabbit anti-Chk1 (Ser345) (#2348, 1: 1000), rabbit anti-Chk2 (Thr68) (#2661, 1: 1000), mouse anti-Chk1(#2360, 1: 1000), rabbit anti-Chk2(#6334, 1: 1000), mouse anti-HDAC1(#5356, 1: 1000) and mouse anti-HDAC2(#5113, 1: 1000) were from Cell Signaling Technology (Danvers, MA, USA); rabbit anti-USP19 (SAB4500170, 1:100 for immunostaining) was from Sigma (St. Louis, MA, USA). MG132 was from Sigma (M7449). Protease inhibitor cocktail was from Roche (04-693-132-001, Mannheim, Germany). Deubiquitinase inhibitor N-ethylmaleamide (NEM) (E3876, Sigma). The siRNAs to target USP19, HDAC1 or HDAC2 were chemosynthesis by Thermo Fisher Scientific. Lipofectamine RNAiMAX Transfection Reagent and Lipofectamine 2000 Reagent (Invitrogen, Carlsbad, CA, USA).

### Cell lines and time-lapse imaging

HCT116 cells were maintained at 37°C in a humidified atmosphere of 5% CO2 in RPMI-1640 medium, supplemented with 10% FBS. HeLa/GFP-H2B stable cell line were maintained at 37°C in a humidified atmosphere of 5% CO2 in DMEM, supplemented with 10% FBS. HeLa/GFP-H2B cells were seeded into an eight-chambered cover glass (Lab-Tek Chambered no 1.0 Borosilicate Cover Glass System, Nunc, Thermo Fisher Scientific Inc., Waltham, MA, USA), 24h later cells were transfected with siRNA and synchronized in mitosis by thymidine block and release. From 72 h after siRNA transfection, images were collected every five minutes using a 0.1-sencond exposure for 12 h using 40×lens objective on inverted fluorescence microscope (Nikon Eclipse Ti-E, Tokyo, Japan) with an UltraView spinning-disc confocal scanner unit (Perkin Elmer, Boston, MA, USA). The temperature of the imaging medium was kept at 37°C. Image sequences were viewed using Volocity software (PerkinElmer Inc., Waltham, MA, USA).

### Immunofluorescence

Cells were fixed with 4% paraformaldehyde for 15 min, followed by permeabilization using PBS with 0.5% Triton X-100 for 10 min at 4°C. Cells were incubated with anti-γH2AX antibody (1:500), anti-ACA antibody (1:200) or anti-USP19 antibody (1:100) at 4°C overnight and further with Alexa Fluor conjugated secondary antibodies (Thermo Fisher Scientific, Grand Island, NY, USA) for 1 hour at room temperature. The DNA was stained by Hoechst 33342 (62249, Thermo Fisher Scientific, Leiden, The Nether-lands). Images were collected by 100×oil objective lens on inverted fluorescence microscope with an UltraView spinning-disc confocal scanner unit.

### Immunoprecipitation

293T cells were transfected as indicated and lysed in M2 buffer (20mM Tris-HCl pH 7.5, 0.5% Nonidet P-40, 250mM NaCl, 3mM EDTA, 3mM EGTA) and protease inhibitor cocktail. The lysates were cleared by centrifugation at 4°C, 12,000 r.p.m. for 20 minute. Supernatants were rotated with antibody overnight and protein G sepharose beads for 2 h at 4°C. The immunocomplexes were washed five times, boiled in sample buffer and subjected to SDS–polyacrylamide gel electrophoresis.

### Ubiquitination analysis

Cell lysates were harvested in RIPA lysis buffer (20mM Tris-HCl pH 7.4, 150mM NaCl, 10mM EDTA, 1% Triton X-100, 1% deoxycholate), NEM and protease inhibitor cocktail. After sonication, cell lysates were centrifuged at 4°C, 12,000 r.p.m. for 15 min twice. Added SDS into the supernatants to a final concentration 1% and boiled at 95°C for 5min in order to remove noncovalently associated proteins. Then the lysates were diluted into 0.1% SDS and immunoprecipitated with Anti-Flag M2 Affinity Gel (A2220, Sigma) for 4 hours. The immunoprecipitants were washed five times with RIPA buffer, boiled in sample buffer, and subjected to SDS–polyacrylamide gel electrophoresis.

### NHEJ/HR-mediated DSB repair assays

EJ5-GFP or DR-GFP U2OS cells were transfected with control or USP19 siRNA using lipofectamine RNAiMAX Transfection Reagent, followed by further transfection with HA-I-SceI or empty vector using Lipofectamine 2000 Reagent. From 72h after vector transfection, cells were collected and analyzed for the proportion of GFP-positive cells by FACS analysis. The NHEJ/HR efficiency presented as relative quantification compared with control siRNA.

### Tissue array and immunohistochemistry

Immunohistochemistry staining for USP19 was performed on the paraffin-embedded kidney clear cell carcinoma tissue array (KD481, KD601; BioMax), followed by secondary antibody and DAB (3, 3’-diaminobenzadine) disclosure. Nuclei were counterstained with hematoxylin. Images were captured using an NanoZoomer Digital Pathology system (Hamamatsu Photonics, Hamamatsu, Japan). USP19 expression levels were semiquantitatively assessed in tissue samples. Both the extent and intensity of USP19 immunostaining were taken into consideration when analyzing the data. The intensity of staining was scored from 0 to 3 and the extent of staining was from 0 to 100%. The final quantitation of each staining was obtained by multiplying the two scores. The slides were analyzed by two independent pathologists.

## SUPPLEMENTARY MATERIALS FIGURE


