# Supplementary material for: USP19 deubiquitinates HDAC1/2 to regulate DNA damage repair and control chromosomal stability
Source: Oncotarget. 2016 Aug 8;8(2):2197–208. doi: 10.18632/oncotarget.11116 (PMC5356792; doi:10.18632/oncotarget.11116)
Supplement: Supplementary file 1 [file oncotarget-08-2197-s001.pdf]

USP19 deubiquitinates HDAC1/2 to regulate DNA damage repair and control chromosomal stability

SUPPLEMENTARY FIGURE

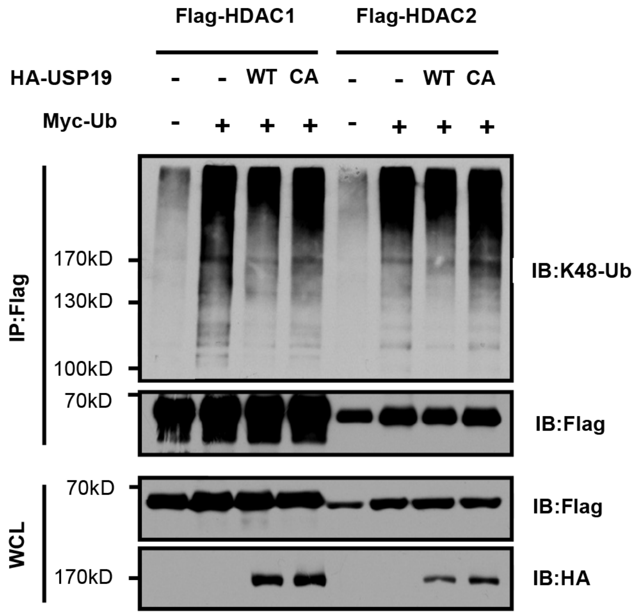

Supplementary Figure S1: HEK293T cells were transfected with Myc-Ub, Flag-HDAC1, Flag-HDAC2, HA-USP19 WT, HA-USP19 CA as indicated, 48h later, treated with MG132 for 4h. Cell lysates were extracted under denaturing conditions (95°C, 1% SDS), then immunoprecipitated with anti-Flag and immunoblotted with linkage-specific antibodies recognizing K48-linked polyubiquitin.
